# *Lyophyllum decastes*-derived polysaccharides alleviate DSS-induced colitis in mice by suppressing inflammation, enhancing intestinal barrier integrity, and restoring gut microbiota homeostasis

**DOI:** 10.3389/fphar.2025.1644325

**Published:** 2025-07-11

**Authors:** Eslam Ghaleb, Yamina Alioui, Jamalat Yazeed, Natheer Wahan, Ahmed Bashah, Ali AL-waqeerah, Aamna Atta, Yuxin Sun, Yi Xin, Liang Wang, Bin Feng, Weifeng Mao

**Affiliations:** ^1^ Biochemistry and Molecular Biology Department, College of Basic Medical Sciences, Dalian Medical University, Dalian, China; ^2^ Bitechnology Department, College of Basic Medical Sciences, Dalian Medical University, Dalian, China; ^3^ Department of Pharmacology, College of Pharmacy, Dalian Medical University, Dalian, China; ^4^ Department of Internal Medicine, The First Affiliated Hospital of Dalian Medical University, Dalian, China; ^5^ National Joint Engineering Laboratory, Stem Cell Clinical Research Center, Regenerative Medicine Center, The First Affiliated Hospital of Dalian Medical University, Dalian, China

**Keywords:** *Lyophyllum decastes*, polysaccharides, ulcerative colitis, dextran sulfate sodium (DSS), intestinal barrier integrity, gut microbiota, anti-inflammatory activity

## Abstract

**Introduction:**

Inflammatory bowel disease (IBD), including ulcerative colitis (UC), is characterized by disturbances in the intestinal barrier, immune system dysfunction, and an altered gut microbiota composition. *Lyophyllum decastes*, a medicinal mushroom known for its bioactive polysaccharides, has shown potential anti-inflammatory properties. However, its therapeutic effects on ulcerative colitis have not been studied. This study investigates the effects of *L*. *decastes* polysaccharides (LDP) on dextran sulfate sodium (DSS)-induced colitis in mice.

**Methods:**

Ulcerative colitis was induced in BALB/c mice by administering 3% DSS in drinking water for 7 days. Mice were then treated orally with LDP at doses of 200 or 400 mg/kg for 14 days. Disease activity index (DAI), histopathological analysis, cytokine levels, myeloperoxidase (MPO) activity, tight junction protein expression (occludin and ZO-1), and gut microbiota composition were assessed.

**Results and discussion:**

LDP treatment significantly reduced DAI scores and preserved colonic histological structure. It modulated cytokine levels, decreasing pro-inflammatory cytokines (TNF-α, IL-6, IL-1β) and increasing anti-inflammatory cytokines (IL-10, IL-4, TGF-β). Additionally, LDP improved intestinal barrier function by reducing MPO activity and enhancing occludin and ZO-1 expression. 16S rRNA sequencing revealed a significant restoration of gut microbiota diversity, with an increase in beneficial bacteria Muribaculaceae*, Lactobacillus,* and Lachnospiraceae, and a reduction in pathogenic bacteria *Escherichia-Shigella*. these findings suggest that LDP exhibits therapeutic effects in DSS-induced colitis through anti-inflammatory properties, enhancement of intestinal barrier function, and modulation of gut microbiota. These findings suggest that LDP may serve as a promising novel therapeutic agent for the management of ulcerative colitis.

## Graphical Abstract

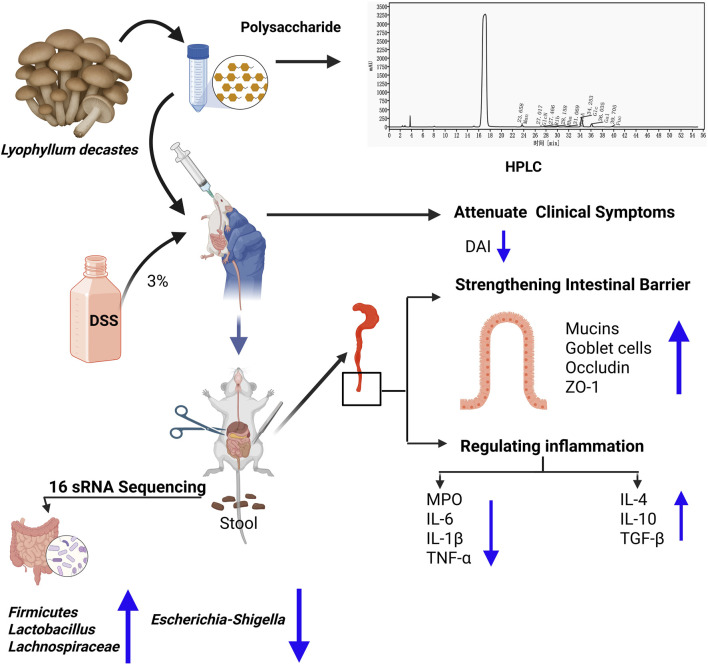

## 1 Introduction

Inflammatory Bowel Disease (IBD) is characterized as a chronic inflammatory disease of the gastrointestinal tract, primarily Ulcerative colitis (UC) and Crohn’s disease (CD). Crohn’s disease may affect any part of the gastrointestinal tract, while UC affects the colonic mucosa. The principal symptoms are diarrhea, abdominal pain, and rectal bleeding (Nakase et al., 2021; Yang et al., 2021). Ulcerative colitis is more prevalent in Western populations, including Europe, Australia, New Zealand, and North America, compared to Asia, Africa, and South America. However, recent studies reveal a rising incidence of UC in China. Approximately 20%–30% of the patients with UC require surgery, which has led the World Health Organization (WHO) to designate UC as a modern refractory disease and a focus of priority study in gastroenterology (Silva et al., 2014; Kotze et al., 2020; Li et al., 2021). The exact etiology of IBD is unknown since there has not been any defined mechanism of its occurrence (Guo H. et al., 2022). Alterations in gut microbiota patterns are accountable for derangement of normal physiological processes and development of intestinal disease, particularly UC. Research has indicated that dysbiosis in UC patients is linked to decreased bacterial diversity, fewer beneficial probiotic species, and higher populations of pathogenic microbes (Alam et al., 2020; Li et al., 2021). The main treatment approach for UC involves aminosalicylates, corticosteroids, and biologic drugs against specific inflammatory pathways (e.g., TNF-α, IL-12/23) to decrease inflammation, preserve remission, and enhance quality of life (Ferretti et al., 2022). Nonetheless, UC cannot be completely cured, and some patients may have unfavorable responses, relapses, complications, or even require surgery. Furthermore, these therapeutic drugs can lead to serious side effects, affecting both gastrointestinal health and liver function (Kennedy et al., 2019). Therefore, there is a growing necessity to determine novel and safe therapeutic approaches for UC (Pan et al., 2022). Polysaccharides are natural macromolecules that play key biological roles and possess bioactive properties, including antioxidant, antidiabetic, anti-inflammatory, antitumor, immunoregulatory, and neuroprotective effects (Gharibzahedi et al., 2022; Chen et al., 2024). *Lyophyllum decastes*, commonly referred to as the “fried chicken mushroom” in Europe due to its texture and flavor, which resembles fried chicken, is known in China as the “pilose antler mushroom.” This name is inspired by the cap’s pattern, which resembles the structure of deer antlers, a symbol of esteemed traditional Chinese medicine. Popular across Asia and Europe, this species has attracted significant attention as a potential functional food ingredient due to its rich content of polysaccharides, proteins, and essential minerals (Li et al., 2025). It has a long history of use as a traditional medicinal mushroom, recognized for its health-promoting properties (Ke et al., 2023). In recent years, *L. decastes* has attracted increasing attention as a functional food due to its rich nutritional content and diverse pharmacological activities, including antitumor effects (Ukawa et al., 2000), lipid-lowering activity (Ukawa et al., 2002), antidiabetic properties (Miura et al., 2002), bacteriostatic action (Arase et al., 2013), hepatoprotective effects (Zhang et al., 2022), obesity-suppressing activities (Wang et al., 2022), and immunomodulatory potential (Ma and Wang, 2023). While *L. decastes* is believed to have potential therapeutic applications, its polysaccharides have never been evaluated in the context of colitis. This study aims to investigate, for the first time, the therapeutic effects of LDP in the treatment of UC, with a particular focus on their influence on gut microbiota composition, intestinal barrier integrity, and inflammatory responses in a DSS-induced colitis model.

## 2 Materials and methods

### 2.1 Chemicals and reagents

Fruiting bodies of *Lyophyllum decastes* were provided by Yunnan Mei Food Co., Ltd. (Yunnan, China). Dextran Sulfate Sodium (DSS) was obtained from Yeasen Biotechnology Co., Ltd. (Shanghai, China). Microbial genomic DNA was extracted from fecal samples using the QIAamp PowerFecal DNA Kit (MoBio Laboratories, Carlsbad, CA, United States). ELIZA assay kits were supplied by Jiangsu Meibiao Biotechnology Co., Ltd. (Jiangsu, China). All primers used in this study were synthesized through Bioengineering Shanghai Co., Ltd. (Shanghai, China). The Primary antibodies (Occludin, Zonula occludens-1 (ZO-1) were purchased from Proteintech (Wuhan, China). Hematoxylin-Eosin (H&E) staining kit (G1120) and AB-PAS Staining Kit (G1285) were purchased from Beijing Solarbio Science & Technology Co., Ltd. (Beijing, China). TRIzol reagent (Thermo Fisher Scientific Waltham, MA, United States). All the other chemicals used in the experiments were of highest analytical grade and purchased commercially.

### 2.2 Extraction of polysaccharides from *L. decastes*

After receiving the fruiting bodies of *L. decastes*, the material was weighed and oven-dried at 45°C until fully desiccated. The dried samples were then ground into a fine powder and sieved through a 0.45 mm mesh to ensure uniform particle size. The powder was extracted with distilled water in a 1:40 (w/v) ratio at 70°C for 3 h in a water bath; this extraction was repeated twice. After filtration, the combined extract was treated with 1.5% (v/v) trichloroacetic acid (TCA) to remove protein impurities. The pH of the solution was adjusted to 7.0 using dropwise addition of sodium hydroxide. The supernatant was then concentrated to one-third of its original volume by rotary evaporation. To induce polysaccharide precipitation, 95% ethanol was slowly added until the final ethanol concentration reached 85% (v/v). The resulting crude polysaccharides were collected by centrifugation at 4,000 rpm for 15 min, washed with ethanol, and vacuum freeze-dried. The dried LDP powder was stored in airtight containers at −20°C until further analysis (Ma and Wang, 2023).

### 2.3 Determination of molecular weight and monosaccharide composition of LDP

Gel permeation chromatography (GPC) was used to ascertain the molecular weight distribution of the crude LDP. The analysis was carried out using an Agilent GPC system that had a refractive index detector and a PL aquagel-OH Mixed-H column (300 mm × 7.5 mm, 8 μm; Agilent Technologies, Santa Clara, CA, United States). At a flow rate of 1.0 mL/min, ultrapure water served as the mobile phase, and the column temperature was kept at 30°C. The calibration curve used to determine the molecular weight parameters of LDP was created using dextran standards with known molecular weights.

For monosaccharide characterization, high-performance liquid chromatography (HPLC) was conducted following acid hydrolysis and PMP (1-phenyl-3-methyl-5-pyrazolone) derivatization. Specifically, 50 mg of purified polysaccharide was hydrolyzed with 2 M trifluoroacetic acid (TFA) at 120°C for 6 h. Excess acid was removed by repeated co-distillation with methanol under reduced pressure. The resulting hydrolysate was dissolved in methanol, neutralized with sodium hydroxide, and incubated at 70°C for 1 h to ensure complete dissolution. Distilled water was then added, followed by a triple-phase extraction with chloroform to eliminate non-polar impurities. The final aqueous layer was filtered through a 0.22 μm nylon membrane filter (Millipore, Westborough, MA, United States) to ensure clarity and sterility. HPLC analysis was carried out using an Agilent 1,260 Infinity II system (Agilent Technologies, Santa Clara, CA, United States) equipped with a ZORBAX Eclipse XDB-C18 column (4.6 × 250 mm, 5 µm) and a UV detector set at 254 nm. The mobile phase consisted of 0.1 M phosphate buffer (pH 6.7) and acetonitrile (82:18, v/v), with a flow rate of 1.0 mL/min. The column temperature was maintained at 30°C, and the injection volume was 20 µL. Monosaccharide standards mannose, ribose, rhamnose, glucose, Galacturonic acid, and fucose were purchased from Sigma-Aldrich (St. Louis, MO, United States) and derivatized under identical conditions.

### 2.4 Animal housing

The study utilized 32 pathogen-free male BALB/c mice (4–6 weeks old) from Dalian Medical University’s SPF breeding facility. Animals were housed in standard laboratory cages under controlled environmental conditions, including a temperature of 22°C ± 2°C, relative humidity of 50 ± 5%, and a 12-h light/dark cycle, for an acclimatization period of 7 days, following approval by the institutional animal ethics committee (Ref: 202410367).

### 2.5 Induction and therapeutic protocol for colitis

**FIGURE 1:**
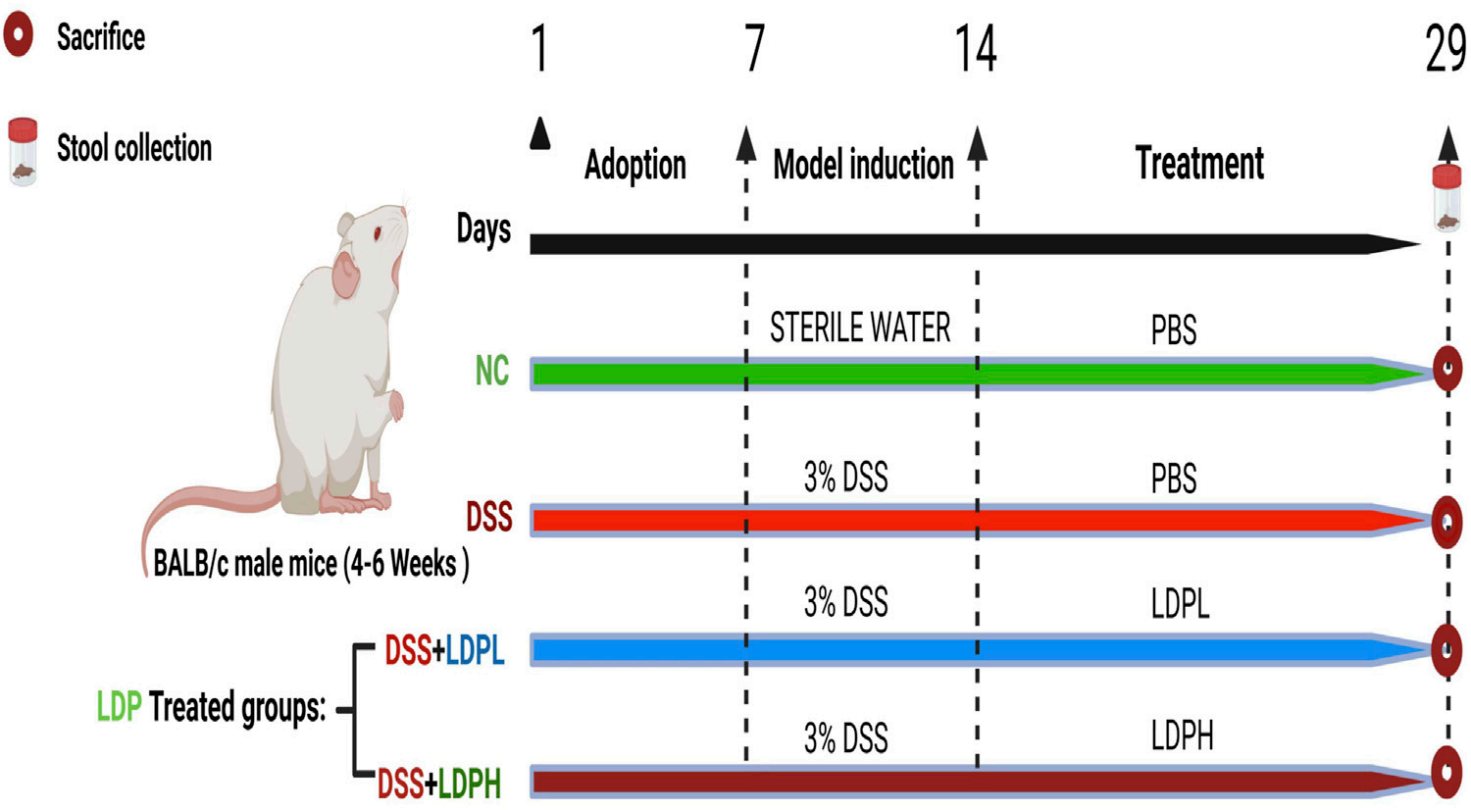
Diagrammatic representation of the experimental timeline and grouping. Mice were divided into four groups (*n* = 8 per group): normal control (NC), DSS-induced colitis model (DSS), low-dose LDP treatment group (LDPL), and high-dose LDP treatment group (LDPH). The NC group received regular drinking water and oral gavage of PBS for 14 days. Colitis was induced in the DSS, LDPL, and LDPH groups by administering 3% DSS in drinking water for 7 days. During and after DSS administration, the DSS group continued receiving PBS by oral gavage, while the LDPL and LDPH groups were treated with LDP at 200 mg/kg/day and 400 mg/kg/day, respectively, for 14 consecutive days. Fecal samples were collected on day 29, and all mice were sacrificed on the same day for subsequent analysis.

Following the adaptation period, mice were randomly allocated into four experimental groups (*n* = 8/group) as shown in Figure 1: (1) Normal control (NC) receiving untreated drinking water; (2) DSS-induced colitis model group; (3) Low-dose *Lyophyllum decastes* polysaccharide treatment (LDPL, 200 mg/kg bw); and (4) High-dose *Lyophyllum decastes* polysaccharide treatment (LDPH, 400 mg/kg bw). Colitis induction was achieved by administering 3% DSS dissolved in sterile water for 7 consecutive days to groups 2–4, while the NC group maintained access to unmodified drinking water throughout the study period. After confirming the induction of UC through clinical symptoms, the LDPL and LDPH groups received daily oral gavage of their respective polysaccharide doses for 14 days. The NC and DSS groups received phosphate-buffered saline (PBS) as a control.

For gut microbiota characterization, fecal samples were collected at termination (day 21 post-intervention) and cryopreserved at −80°C to ensure microbial genomic stability during storage. After the experimental period, all animals were euthanized for tissue collection. Following euthanasia, the colon, small intestine, spleen, liver, and thymus were surgically excised. Colonic segments were immersion-fixed in 4% phosphate-buffered formalin for histopathological assessment, while all other tissues were immediately flash-frozen in liquid nitrogen and stored at −80°C for subsequent biomolecular investigations. This sampling strategy facilitated the evaluation of localized intestinal pathology and systemic treatment effects.

### 2.6 Evaluation of disease activity index

Throughout the experimental period, the severity of colitis was monitored daily using the Disease Activity Index (DAI), as originally proposed by Murthy et al. and later refined in subsequent studies as Yang et al. (Murthy et al., 1993; Yang et al., 2024). The DAI combines three clinical parameters, each scored on a scale of 0–4: (1) Body weight loss (0 = none; 1 = 1%–5%; 2 = 5%–10%; 3 = 10%–15%; 4 = >15%), (2) Stool consistency (0 = normal; 1–2 = loose stools; 3–4 = watery diarrhea), and (3) Rectal bleeding (0 = none; 1 = occult blood; 2 = positive occult blood; 3 = gross bleeding). The overall DAI was calculated as the average of the three scores using the formula:

$$\text{DAI}=\left(\text{Weight loss score}+\text{Stool consistency score}+\text{Bleeding score}\right)\;/\;3$$

### 2.7 Assessment of body weight and organ indices

Throughout the experimental period, Animal body weights were measured and recorded daily using a calibrated electronic balance (±0.1 g precision) to monitor physiological changes and treatment effects, while daily measurements of food and water consumption were conducted. Following euthanasia, the total colonic length was determined for each subject. Additionally, the organ index was computed for key organs, including the colon, small intestine, spleen, and thymus, using the standardized formula:

$$\text{Organ Index }\left(\text{mg}/g\right)=\text{Organ Weight }\left(\text{mg}\right)\;/\text{ Body Weight }\left(g\right)$$

### 2.8 Histopathological analysis of colonic tissue

Following euthanasia, colonic segments were promptly dissected and immersion-fixed in 4% neutral-buffered formalin (pH 7.4) at ambient temperature. Processed tissues were dehydrated in graded alcohols, cleared in xylene, and paraffin-embedded. Four-micron sections were stained with hematoxylin and eosin, and examined by two independent, blinded observers using a Leica DM750 microscope (Leica Biosystems, Wetzlar, Germany). In this study, histological evaluation of colon samples was conducted based on two primary parameters: regeneration and inflammation. The regeneration score ranged from 0 to 4, with a score of 4 indicating the absence of tissue repair, while a score of 3 denoted disrupted surface epithelium. A score of 2 represented ongoing regeneration accompanied by crypt depletion, whereas a score of 1 reflected near-complete regeneration. A score of 0 corresponded to complete tissue regeneration or the presence of normal histological architecture. Inflammation was scored independently on a scale from 0 to 3, with 3 signifying severe inflammation, 2 indicating moderate inflammation, 1 reflecting slight inflammatory infiltration, and 0 representing the absence of inflammation. This scoring system facilitated the quantification of histopathological changes for comparative analysis of tissue responses under different experimental conditions (Alioui et al., 2024).

### 2.9 Evaluation of mucin production, goblet cell density, and mucus layer thickness

To evaluate Mucin-2 expression in colon tissue, immunohistochemistry (IHC) was conducted. 5 μm paraffin-embedded colon tissue sections were mounted on positively charged slides. These slides were deparaffinized in xylene and rehydrated using a graded ethanol series, following the protocol of the SP-KIT9720 immunohistochemical staining kit (MXB Biotechnologies, Beijing, China), as per the manufacturer’s instructions. A semi-quantitative approach was employed for data analysis, with each slide being examined three times at random in different fields to assess the immunolabeled cells. Periodic Acid-Schiff (PAS) staining was performed to assess goblet cell distribution and mucous epithelial thickness of colonic tissues. Tissue sections were deparaffinized in xylene and rehydrated using a graded ethanol series first. Samples were oxidized in 0.5% periodic acid solution (5 min, RT), washed with distilled water, and incubated with Schiff’s reagent (7 min, RT, dark). Following a 10–15 min wash with running water, sections were counterstained in Mayer’s hematoxylin, washed in tap water (10 min), and dehydrated through a graded series of ethanol. Slides were air-dried, then coverslipped with mounting medium. Alcian Blue staining (AB) at pH 2.5 was employed to selectively identify epithelial acid mucins, including both non-sulfated and sulfated forms. Initially, slides were deparaffinized and hydrated as previously described. The slides were stained by immersion in 1% aqueous alizarin blue acetate for 10 min, followed by three washes for 6 min each. Oxidation was performed using 1% periodic acid solution for 5 min, followed by two washes with ultra-pure water, 6 min each. Schiff’s solution was applied for 10 min for further staining, and the slides were washed in tap water for 10 min. Finally, the slides were dehydrated with a series of ascending alcohol solutions, clarified, and mounted. Image capture was done using a Leica DM750 microscope (Leica Microsystems, Wetzlar, Germany). Blinded histopathological analysis by an independent researcher provided an unbiased assessment. ImageJ software was used for semi-quantitative analysis.

### 2.10 Immunofluorescent analysis of tight junction protein

To enable immunofluorescence detection of Occludin and ZO-1, tight junction-associated proteins, and nuclear staining, the slides underwent an initial heating process at 65°C for 2 h to improve tissue adherence. This was subsequently achieved by deparaffinization using xylene. After the initial steps, rehydration was performed by immersing the samples in a series of ethanol solutions with concentrations of 100%, 95%, and 70%, followed by a rinse with ultrapure water. Antigen retrieval was conducted by immersing the tissue sections in a citrate buffer (10 mM, pH 6.0) and heating them in a microwave oven. This was followed by three rinses with PBS. The sections were then blocked with 5% BSA in PBS and left to incubate overnight at 4°C with primary antibodies: anti-ZO-1 (1:1000, Proteintech Group, Inc., Wuhan, China; 21773-1-AP) and anti-Occludin (1:400, Proteintech Group, Inc., Wuhan, China; 27260-1-AP). After the primary antibodies were applied, the slides underwent PBS washes and were then treated with FITC-conjugated Affinipure goat anti-rabbit secondary antibody (Proteintech Group, Inc., Wuhan, China) for 1 h at room temperature. Following further PBS washes, DAPI was used to stain nuclei for 5 min. Finally, the slides were mounted and examined under a fluorescence microscope.

### 2.11 Assay of MPO activity and inflammatory cytokines in colon tissue

To assess myeloperoxidase (MPO) enzymatic activity and inflammatory cytokines related to ulcerative colitis within colonic tissues, 100 mg of fresh tissue was homogenized in 900 µL of ice-cold PBS. The homogenates were centrifuged at 3,500 rpm for 15 min at 4°C to separate cellular debris. The resulting supernatant was carefully collected for subsequent biochemical evaluation. MPO activity in the intestinal samples was quantified utilizing a commercially available ELISA kit (Jiangsu Meibiao Biotechnology Co., Ltd., China). Before assay setup, all reagents were equilibrated to room temperature for 30 min, following the manufacturer’s instructions. Standard solutions (50 µL) were dispensed into the designated calibration wells, while test samples were aliquoted into the corresponding assay wells. An HRP-conjugated reagent (100 µL) was added to all wells, and the microplate was incubated at 37°C for 1 h to allow immunological interactions. Post-incubation, the wells were washed five times using the supplied washing buffer to remove unbound materials. To initiate the colorimetric reaction, 50 µL each of chromogen solutions A and B was added sequentially to each well, followed by a 15-min incubation at 37°C in the dark. The reaction was terminated by the addition of 50 µL stop solution, and absorbance was measured at 450 nm within 15 min. A standard curve was generated from the known concentrations, and MPO levels and inflammatory cytokines in the samples were extrapolated accordingly.

### 2.12 Quantitative real-time PCR analysis of inflammatory gene expression

To investigate the regulation of inflammatory mediators at the gene level, we performed quantitative real-time PCR (qRT-PCR) on colon tissue samples from all experimental groups. Approximately 50 mg of colon tissue was collected, and total RNA was extracted using TRIzol reagent (Thermo Fisher Scientific Waltham, MA, United States). RNA quality and concentration were assessed using a NanoDrop One spectrophotometer (Thermo Fisher Scientific, United States) and verified by agarose gel electrophoresis. First-strand cDNA was synthesized from 1 µg of total RNA using HiScript II Q RT SuperMix (Vazyme Biotech, China), following the manufacturer’s protocol. qRT-PCR was performed in 10 µL reaction volumes using SYBR Green Master Mix (Vazyme Biotech, China) and gene-specific primers, as listed in Supplementary Table S2. Amplification was carried out in the Bioer Light gene 9600 analyzer by Hitech (Beijing, Hangzhou, 310053, China) under the following thermal cycling conditions: initial denaturation at 95°C for 30 s, followed by 40 cycles of denaturation at 95°C for 5 s and annealing at 60°C for 30 s, concluding with melt curve analysis from 65°C to 95°C. The expression levels of IL-6, TNF-α, IL-10, and TGF-β were quantified, with β-actin serving as the internal control. Primer sequences are provided in Supplementary Table S2. All reactions were run in triplicate, and relative expression was calculated using the 2^–ΔΔCt^ method.

### 2.13 Isolation and 16S rRNA-Based pyrosequencing of gut microbial genomic DNA

Genomic DNA was extracted from fecal samples using the PowerMax DNA Isolation Kit (MoBio Laboratories, Carlsbad, CA, United States), following the manufacturer’s protocol. Briefly, 200–500 mg of fecal matter was homogenized, and bacterial cell walls were lysed using lysozyme and chaotropic agents. Proteins were digested with protease, and DNA was purified through silica-based spin-column centrifugation. The purified DNA was eluted, quantified using a NanoDrop ND-1000 spectrophotometer (Thermo Fisher Scientific, United States), and assessed for integrity by 1% agarose gel electrophoresis. Samples were stored at −80°C until further analysis.

The hypervariable V3–V4 regions of the bacterial 16S rRNA gene were amplified using primers 341F and 518R. The PCR program included an initial denaturation at 98°C for 30 s, followed by 25 cycles of denaturation at 98°C for 15 s, annealing at 58°C for 15 s, and extension at 72°C for 15 s, with a final extension at 72°C for 1 min. Amplicons were sequenced using the Illumina NovaSeq 6000 platform (Sangon Biotech Co., Ltd., Shanghai, China). Raw sequencing data were analyzed using QIIME software. Alpha diversity indices (Chao, Shannon, ACE, Simpson) were calculated to assess species richness and diversity. Beta diversity was evaluated using principal coordinates analysis (PCoA), principal component analysis (PCA), and non-metric multidimensional scaling (NMDS) based on weighted UniFrac distances. LEfSe analysis with linear discriminant analysis (LDA) was used to identify differentially abundant taxa and microbial biomarkers among groups.

### 2.14 Statistical analysis

Prism 9 was used to conduct the statistical analysis for this study. Ordinary analysis of variance (ANOVA) followed by Tukey’s multiple comparison tests was used to compare more than two groups with equal standard deviation. A p-value less than 0.05 was considered statistically significant, and all data were presented as mean standard deviation (SD). Furthermore, data relating to 16s rRNA sequencing. A Kruskal–Wallis and Wilcoxon test was used for LEfSe analyses, while Mann-Whitney tests were used for phenotype and OUT analyses.

## 3 Results

### 3.1 Monosaccharide and molecular weight characterization of LDP

**FIGURE 2:**
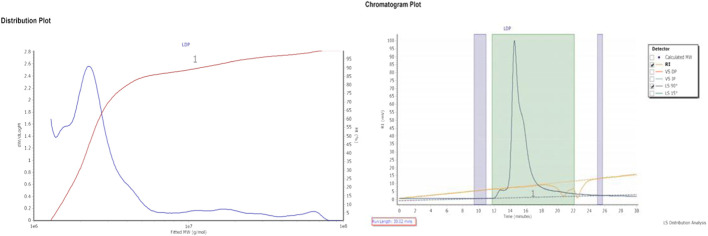
Molecular weight distribution of LDP obtained through gel permeation chromatography.

The polysaccharide yield from *L. decastes* was determined to be 18% (w/w), containing 1.4% protein and 59 mg/mL total carbohydrates (D-glucose equivalent). HPLC analysis of the crude LDP polysaccharide revealed a heteropolysaccharide composition consisting of several monosaccharides, as represented in Table 1. Furthermore, gel permeation chromatography analysis (Figure 2) revealed that the crude LDP exhibited a molecular weight (Mw) of approximately 5.59 × 10^6^ Da, and a number-average molecular weight (MN) of 2.47 × 10^6^ Da, indicating that it is a high-molecular-weight polysaccharide. This considerable molecular size implies a complex structural arrangement, which may contribute to its notable biological activities, including immunomodulatory and anti-inflammatory effects.

**TABLE 1:** Illustrates the composition of the LDP polysaccharide.

Components	Concentration (mg/kg)	Percentage (%)	Retention time (min)
Glucose	154961.5	67.02823	34.17
Galactose	36376.92	15.73475	35.97
Mannose	23434.62	10.13659	23.733
Fucose	9153.85	3.959475	39.66
Ribose	5984.62	2.588633	27.52
Galacturonic acid	1276.92	0.552329	29.94

### 3.2 LDP attenuates inflammatory symptoms in DSS-induced colitis

**FIGURE 3:**
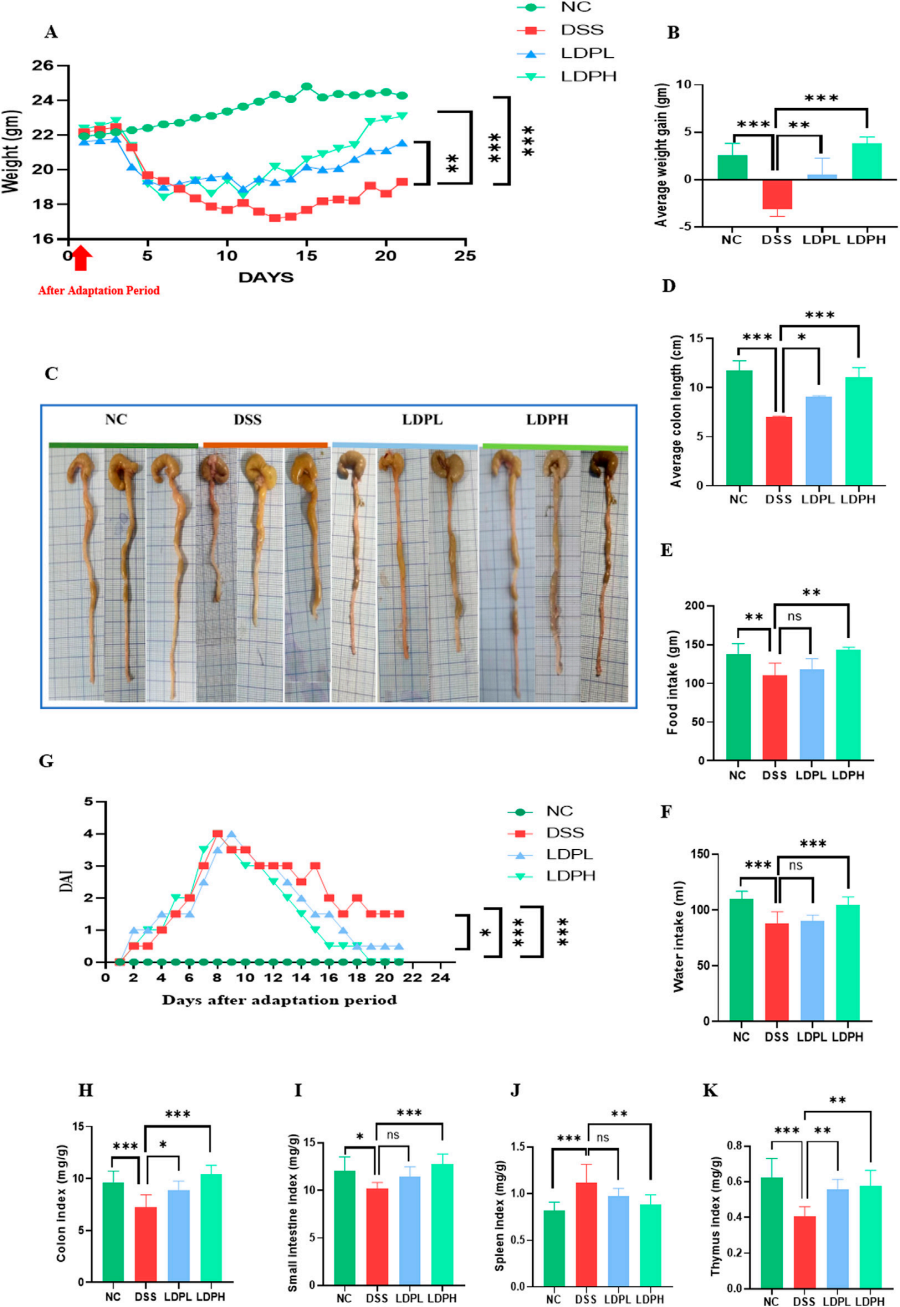
LDP alleviate the clinical symptoms of colitis **(A)** Curve of average weight change **(B)** Average weight gain **(C)** Length of colon **(D)** Average colon length **(E)** Food intake; **(F)** Water intake **(G)** Disease Activity Index **(H)** Colon index **(I)** Small intestine index **(J)** Spleen index **(K)** Thymus index. All data are presented as mean ± SD. Statistical significance is indicated as follows: ns, not significant; *p < 0.05, **p < 0.001, ****p < 0.0001.

After administration of DSS in drinking water, all mice were closely monitored daily to track changes in body weight. As shown in Figures 3A,B,G, a decrease in body weight accompanied by bloody diarrhea and rectal bleeding was observed in all groups that received DSS during the modeling period. Together, these symptoms confirmed the success of inducing an ulcerative colitis model using DSS.

During the recovery period, weight regain was observed in all mice (Figure 3B). However, the weight gain of the DSS group was significantly less compared to the normal control group (p < 0.001). In contrast, following the administration of LDP, both the LDPL and LDPH treatment groups showed a notable increase in weight, and this increase was significantly greater relative to the DSS group (p < 0.01, p < 0.001, respectively). DSS administration also negatively affected food and water consumption in the model DSS group, as shown in Figures 3E,F. DSS administration decreased the consumption of food and water in the DSS model group. However, treatment with LDP improved and increased food and water intake. As shown in Figures 3C,D, the model group experienced a significant decrease in colon length compared to the NC group (p < 0.001). Remarkably, administration of LDP facilitated the restoration of colon length significantly in both the LDPL (p < 0.05) and LDPH (p < 0.001) groups. Beyond these observations, we calculated organ indices, including those of the colon, small intestine, thymus, and spleen, which serve as a critical metric to assess the severity of inflammation. The results indicated a significant decrease in the thymus, small intestine, and colon indices were observed in the DSS group compared to the normal control group, while a significant increase was noted in the spleen index. Thymic atrophy and splenomegaly are known to be key indicators of impaired immune responses typically associated with inflammatory conditions. Crucially, the aforementioned pathological alterations exhibited significant improvement following treatment with LDP in both LDPL and LDPH groups, as visually represented in Figures 3H–K. Furthermore, LDP demonstrated a clear dose-dependent protective effect. Specifically, LDP treatment resulted in a significant suppression of splenomegaly (abnormal enlargement of the spleen) and thymic atrophy (shrinkage of the thymus) in a manner that correlated with the administered dose, compared to the control model group treated with DSS. These findings strongly suggest that LDP possesses significant immunomodulatory activities, which likely play a key role in the observed therapeutic benefits of LDP in the context of colitis, a type of inflammatory bowel disease.

### 3.3 LDP enhancing DAI in DSS-induced colitis

To evaluate the severity of ulcerative colitis, DAI was employed, and the corresponding results are illustrated in Figure 3G. The data revealed a marked increase in DAI scores in all groups that were administered DSS, including the LDPL and LDPH treatment groups. This increase is indicative of the development of typical ulcerative colitis symptoms, such as weight loss, the presence of blood in the stool, and diarrhea. Conversely, the normal control group consistently maintained a DAI score of zero throughout the experimental period, signifying the absence of disease. Notably, following the cessation of DSS administration and the commencement of LDP treatment, the treated groups exhibited significant improvements compared to the DSS control group. Specifically, the LDPH group demonstrated a substantial decrease in DAI scores, eventually reaching zero by the conclusion of the trial, thus indicating a significant alleviation of disease symptoms. On the last day of the experiment, statistical analysis revealed notable differences in DAI scores between the DSS group and the NC group (p < 001), as well as between the LDPL group and the DSS group (p < 0.05), and between the LDPH group and the DSS group (p < 0.001). These statistically significant findings strongly support the therapeutic efficacy of LDP treatment, particularly at the higher dose, in mitigating the severity of DSS-induced ulcerative colitis, as evidenced by the significant reduction in DAI scores.

### 3.4 LDP improves histological changes and attenuates DSS-induced colon injury in mice

**FIGURE 4:**
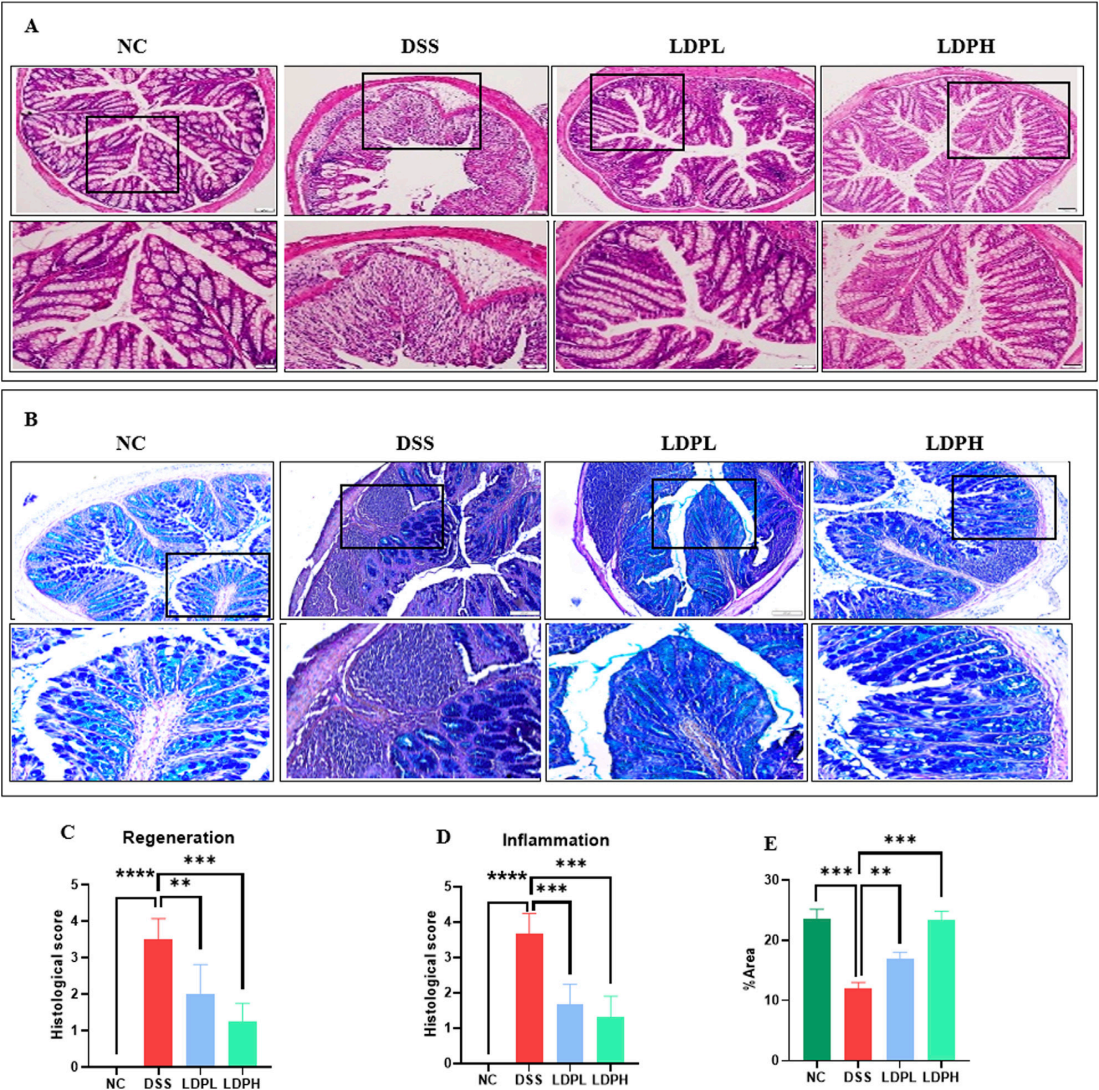
LDP attenuates histopathological damage in DSS-induced colitis. **(A)** Representative images of colonic tissue stained with H&E illustrating the protective effects of LDP against DSS-induced mucosal injury. Magnifications: upper panels ×10, lower panels ×20. **(B)** Alcian Blue staining highlights acid mucins produced by goblet cells as an indicator of epithelial integrity. Magnification panels ×10, ×20. **(C,D)** Quantitative histological scoring for epithelial regeneration **(C)** and inflammatory infiltration **(D)**. **(E)** Quantification of Alcian Blue–positive area, reflecting goblet cell activity and mucin content. All data are presented as mean ± SD. Statistical significance is indicated as follows: ns, not significant; *p < 0.05, **p < 0.001, ****p < 0.0001.

To assess the effect of LDP on histopathological changes in UC murine models, tissues of the colon were observed with H&E staining procedures. As indicated in Figures 4A,C,D, the NC group’s colonic mucosa was well preserved, with normal crypts and intact intestinal gland epithelial cells. No ulceration and negligible inflammatory cell infiltration were noted. Conversely, the model DSS group showed widespread injury to the basic architecture of colonic tissues, as indicated by a decrease in the mucosal epithelial thickness, massive infiltration of neutrophils, and prominent inflammatory cell presence in the mucosal, submucosal, and muscular layers. In addition, the colon had extensive ulceration and submucosal edema. Nevertheless, following LDP treatment, the colon mucosa was observed to be comparatively intact, with orderly glands, slight edema, less ulceration, and lower inflammatory cell infiltration in ulcerative colitis mice. The results indicate the effectiveness of LDP in protecting against histopathological alterations in DSS-induced ulcerative colitis. The result implies that LDP treatment considerably alleviates structural impairment and inflammatory reactions, and it further indicates its potential as a treatment strategy.

### 3.5 LDP promotes epithelial repair by increasing the expression of mucins

**FIGURE 5:**
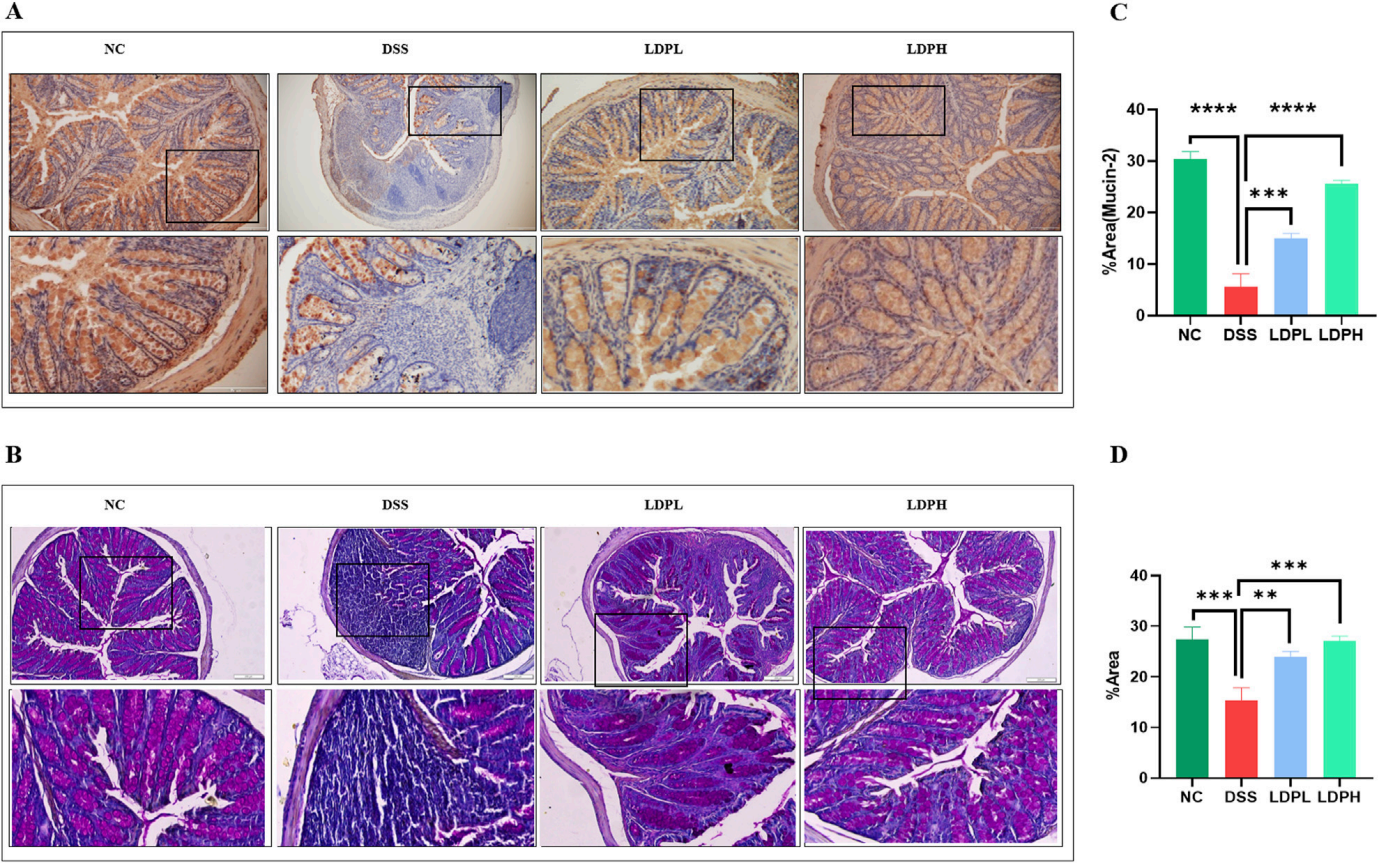
LDP enhances mucin production in the colon. **(A)** Immunohistochemical staining illustrating Mucin-2 expression in colonic tissues across experimental groups. **(B)** Periodic Acid–Schiff staining indicates neutral mucin production by goblet cells. **(C)** Quantitative analysis of Mucin-2 expression based on IHC staining. **(D)** Quantification of PAS-positive goblet cells reflecting neutral mucin content. All data are presented as mean ± SD. Statistical significance is denoted as: **p < 0.01, ***p < 0.001, ****p < 0.0001.

The protective barrier of the colonic epithelium is the intestinal mucus layer and consists of acidic and neutral mucin secreted by goblet cells. To evaluate the integrity of this layer, goblet cell production, and mucin expression, Alcian blue (Figures 4B,E) and Periodic Acid Schiff (Figures 5B,D) staining techniques were used. In addition, immunohistochemistry (Figures 5A,C) was used to identify the expression of Mucin-2, one of the major mucins secreted by goblet cells into the colonic lumen. The results of PAS and AB showed severe intestinal barrier injury in the model group, as indicated by the thinned mucus layer, reduction of goblet cell number, and decreased production of mucus compared with the normal control group. In contrast, LDP treatment notably increased goblet cell formation and mucin expression, such that mucus layer thickness in the LDPL and LDPH groups was significantly recovered. That which was observed in the polysaccharide treatment groups was very similar to that of the normal control group, suggesting LDP promotes the restoration of the mucus layer of DSS-treated mice.

### 3.6 LDP strengthens mucosal barrier function through modulation of tight junction components

**FIGURE 6:**
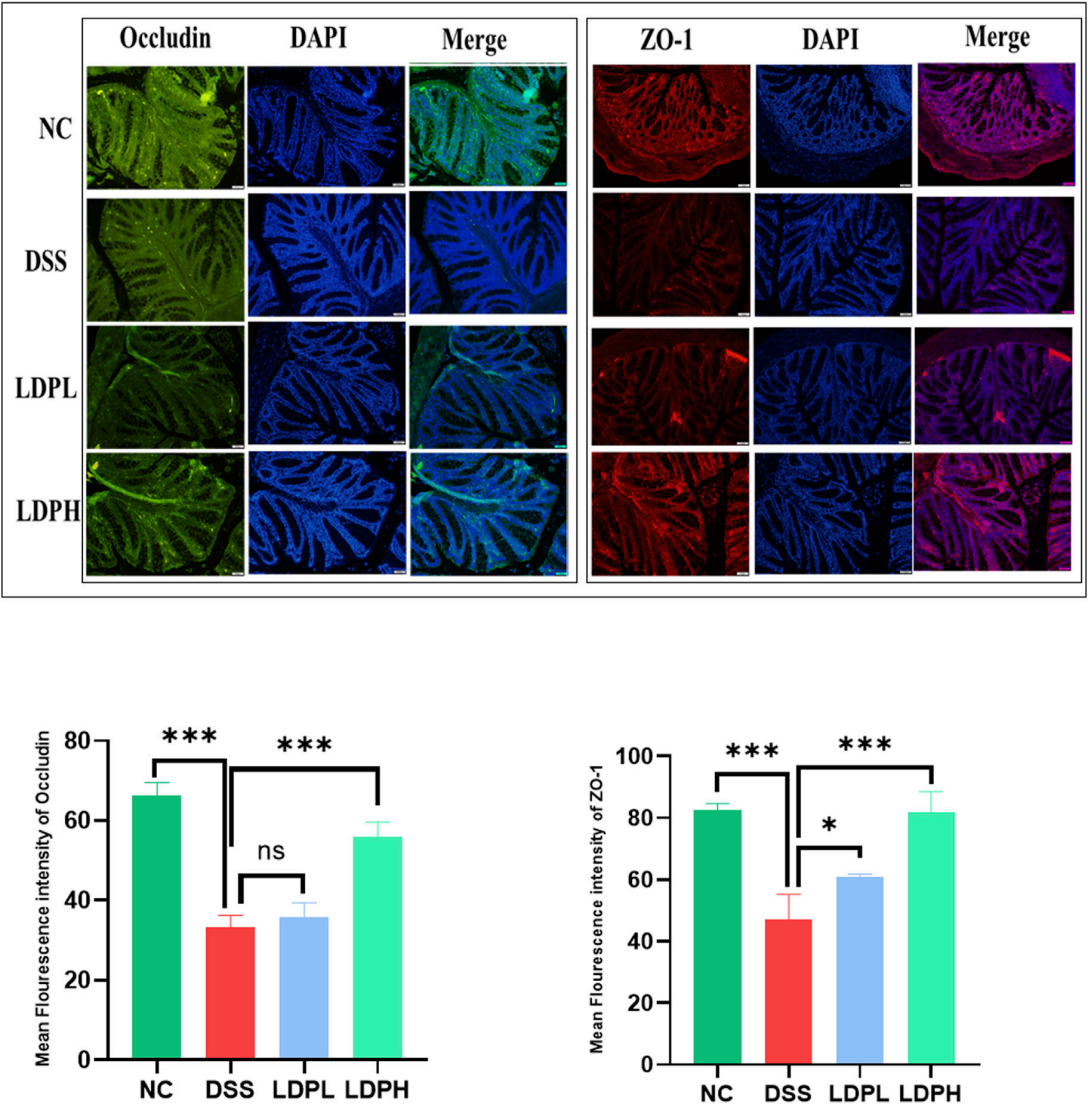
Immunofluorescent (IF) analysis demonstrates that LDP enhances Colonic expression of the tight junction proteins Occludin and ZO-1 (×20 magnification). Quantitative analysis of IF staining intensity is presented alongside statistical significance (ns: not significant; *p < 0.05, **p < 0.01, ***p < 0.001).

To evaluate the impact of therapeutic LDP on tight junction proteins and preserve the integrity of the intestinal barrier, immunofluorescence staining analysis was conducted. Colitis is widely recognized for impairing colonic barrier function, leading to heightened intestinal permeability. As depicted in Figure 6, DSS caused a significant reduction in the levels of ZO-1 and Occludin proteins in the colon tissue of the model DSS group. In contrast, treatment with LDP effectively counteracted this reduction in both LDPL and LDPH. These findings underscore the pivotal role of LDP in safeguarding the intestinal barrier during colitis. Moreover, the ability of LDP to upregulate tight junction proteins, including ZO-1 and Occludin, suggests a potential mechanism by which it could exert its therapeutic effects in ulcerative colitis. This highlights the promising therapeutic potential of LDP in restoring intestinal integrity and mitigating the effects of ulcerative colitis.

### 3.7 LDP regulates the expression of cytokine mRNA in the colon tissue

**FIGURE 7:**
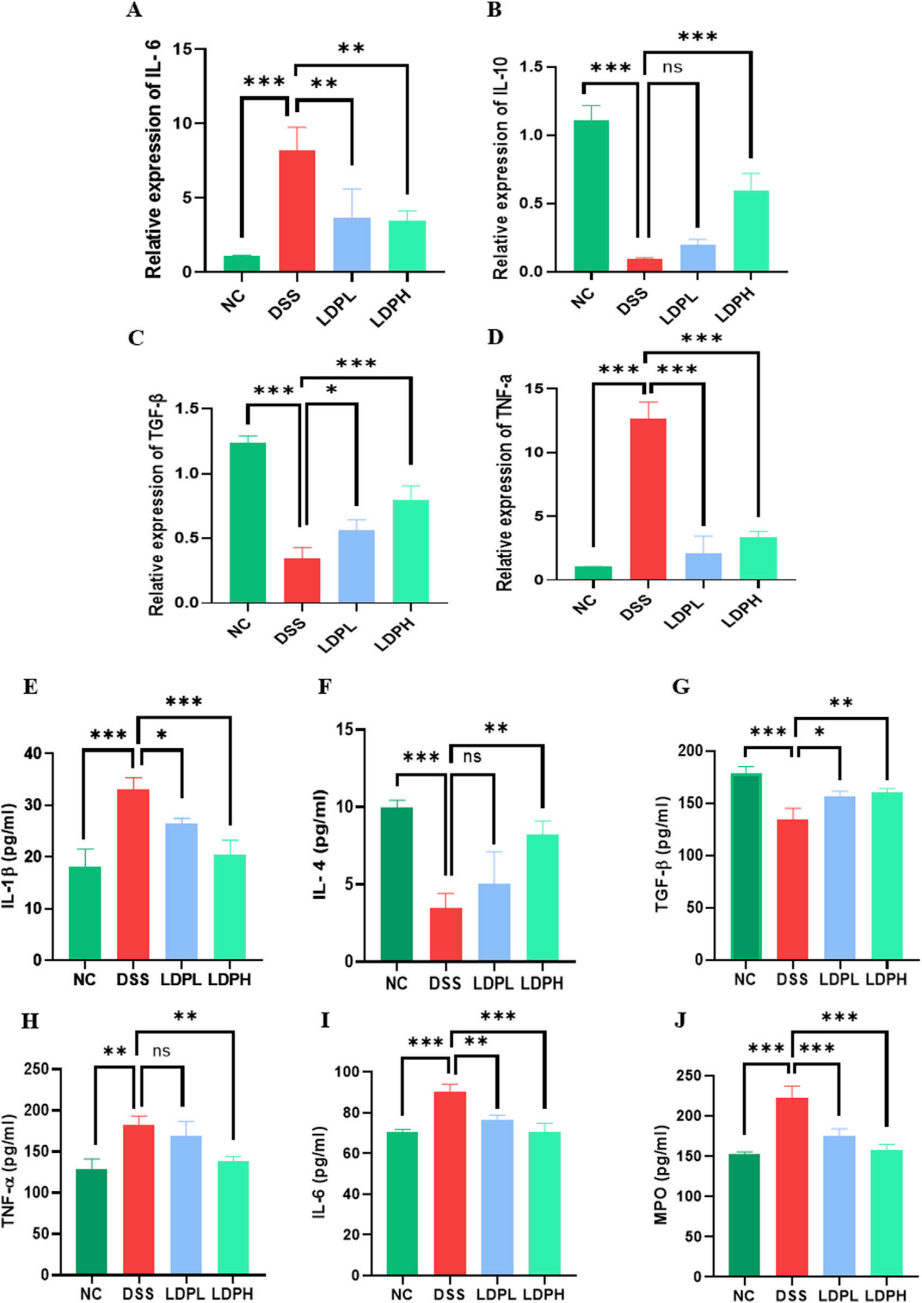
The effect of LDP on pro- and anti-inflammatory cytokine expression and secretion in colon tissue. **(A–D)** Illustrate relative IL-6, IL-10, TGF-β, and TNF-α mRNA expression levels. **(E–J)** Depict IL-1β, IL-4, TGF-β, TNF-α, IL-6, and MPO concentrations in colon tissue by ELISA measurements. All data are presented as mean ± SD. Statistical significance is indicated as follows: ns, not significant; *p < 0.05, **p < 0.001, ****p < 0.0001.

As shown in Figures 7A–D, DSS treatment significantly increased the expression of pro-inflammatory cytokines IL-6 and TNF-α in the DSS model group (p < 0.001). IL-6 levels increased significantly in the DSS group (p < 0.001), while TNF-α expression showed an even greater increase (p < 0.001). Administration of LDP at both lower and higher doses markedly reduced the expression of these cytokines. Notably, TNF-α expression was significantly reduced in both LDPL and LDPH groups compared to DSS (p < 0.001), indicating a robust anti-inflammatory effect. Conversely, the expression of anti-inflammatory cytokines IL-10 and TGF-β was significantly downregulated in the DSS group compared to NC (p < 0.001 for both). Treatment in both low and high doses induced the re-establishment of cytokine expression in a dose-dependent manner. Notably, IL-10 levels were significantly higher in the LDPH group compared to the DSS group (p < 0.001), with no statistical difference for the LDPL group (ns). In the same way, TGF-β expression was significantly higher in both treated groups compared to DSS (p < 0.001 for LDPH, p < 0.05 for LDPL). These results demonstrate that LDP treatment reverses DSS-induced inflammation by downregulating pro-inflammatory markers and upregulating anti-inflammatory cytokines, showing promise as a drug therapy for inflammatory bowel disease.

### 3.8 LDP reduces colonic inflammation through cytokine modulation

To further elucidate the modulatory impact of LDP on cytokine release, ELISA was used to measure protein concentrations of IL-1β, IL-6, IL-4, TGF-β, and TNF-α in colonic tissue homogenates. As presented in Figures 7E–I, the IL-1β level in the DSS group was remarkably higher than that of the NC group (p < 0.001), suggesting a rigorous inflammatory response. Treatment with LDP led to a significant reduction in IL-1β concentrations, particularly in the high-dose group, which restored IL-1β levels closer to those observed in the NC group (p < 0.001 vs. DSS). IL-6, another pro-inflammatory cytokine, was significantly upregulated in DSS mice (p < 0.001), and both LDPH and LDPL treatments significantly suppressed its expression (p < 0.001 and p < 0.01, respectively). For IL-4, which plays a regulatory and anti-inflammatory role, its levels were significantly decreased in the DSS group (p < 0.001). The LDPH group showed a significant increase in IL-4 levels compared to DSS (p < 0.01), while the LDPL group did not differ significantly from DSS (ns), indicating a dose-dependent modulatory effect of LDP. The secretion of TGF-β, a key anti-inflammatory and tissue-repair cytokine, was significantly reduced in the DSS group compared to NC (p < 0.001). Both LDP-treated groups exhibited significantly higher levels of TGF-β compared to DSS (p < 0.05 for LDPL; p < 0.01 for LDPH), suggesting partial restoration of regulatory pathways. Conversely, TNF-α levels were significantly increased in the DSS group (p < 0.01), confirming the pro-inflammatory status induced by DSS. While the LDPL group showed no significant reduction in TNF-α compared to DSS (ns), the LDPH group exhibited a statistically significant decrease (p < 0.01), further supporting the anti-inflammatory effect of higher LDP doses. These ELISA results corroborate the gene expression data, collectively indicating that LDP treatment attenuates DSS-induced inflammation by downregulating pro-inflammatory cytokines (IL-1β, IL-6, TNF-α) and upregulating anti-inflammatory markers (TGF-β, IL-4), especially at higher doses.

### 3.9 Effect of LDP on neutrophil infiltration

As shown in Figure 7J, colon tissue from the DSS group had a significantly higher MPO activity compared to the NC group (p < 0.001). On the other hand, the administration of LDP significantly decreased MPO levels in colonic tissue from both the LDPH and LDPL treatment groups, bringing them close to the normal levels observed in the NC group (p < 0.001 for both). These results suggest that LDP may have an inhibitory effect on neutrophil accumulation, as evidenced by the reduced MPO activity.

### 3.10 Effects of LDP on the diversity of microbial communities in ulcerative colitis mice

**FIGURE 8:**
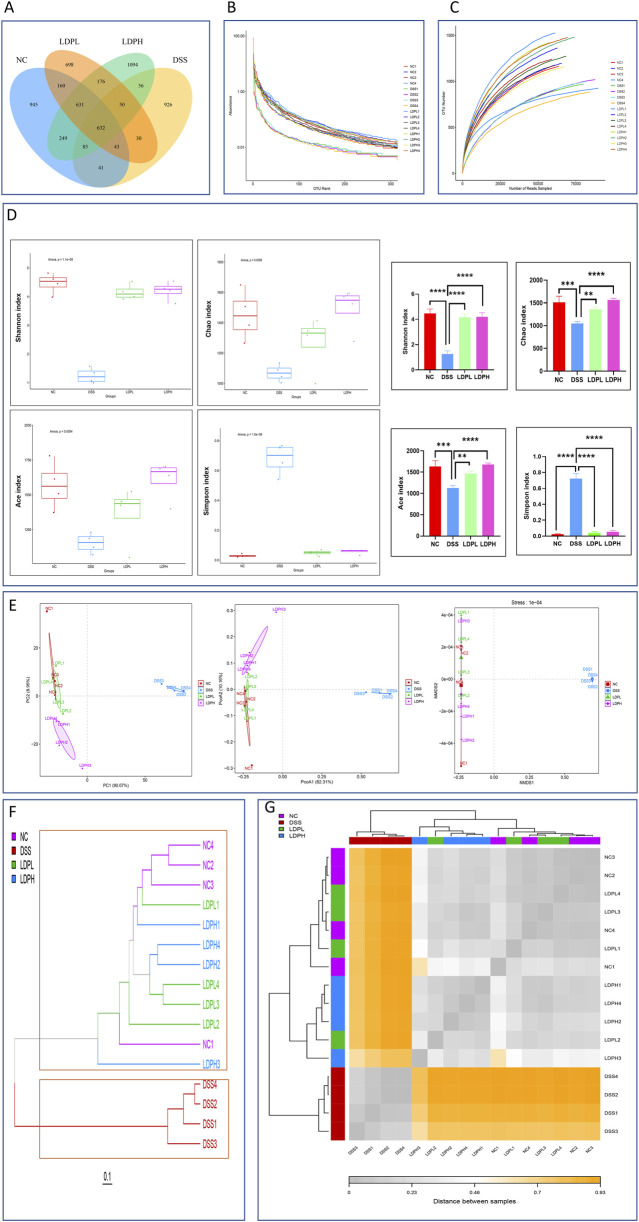
LDP ameliorates DSS-induced gut dysbiosis through microbial community modulation **(A)** Venn diagram of operational taxonomic units (OTUs) demonstrating shared microbiota across experimental groups. **(B)** Rarefaction curve analysis evaluating sampling depth and microbial diversity. **(C)** Rank-abundance distribution showing relative taxonomic abundance patterns. **(D)** α-Diversity indices (Chao1, Simpson, Shannon, ACE) quantifying community richness and evenness. With graphs show the significant effect of LDP on the alpha diversity indexes. **(E)** β-Diversity assessment through multivariate analyses: principal coordinates analysis (PCoA), principal component analysis (PCA), and non-metric multidimensional scaling (NMDS). **(F)** β-diversity assessed by the cluster tree **(G)** heatmap analysis.

Alpha and beta diversity analyses were conducted for each group to evaluate bacterial richness, abundance, diversity, and structural alterations. As shown in (Figure 8A), venn diagram analysis was conducted based on Operational Taxonomic Units (OTUs). The analysis revealed substantial differences in microbial diversity across the groups. The NC group exhibited the highest level of microbial diversity, with a count of 2,831 OTUs, which demonstrates the intricacy of a healthy gut microbiome. However, the DSS-induced colitis model group exhibited a dramatic reduction in OTU count (1,863), which demonstrates a considerable loss of microbial diversity that is generally linked with inflammation and dysbiosis. Upon treatment using LDP extract, there was a rise in OTU counts for both treatment groups. The LDPL group had a total of 2,420 OTUs, indicating a modest restoration of microbial richness. Importantly, the LDPH group had the highest OTU number among the groups, with a total of 3,081, which was even greater than that of the healthy control group. This result indicated that high-dose treatment can not only ameliorate dysbiosis induced by DSS but also lead to an increase in overall microbial diversity, which may promote gut health and resilience. The findings emphasize the varied impact of inflammatory states and therapeutic interventions on gut microbiota composition, thus enhancing the capacity of LDP therapies to permit the reestablishment of microbiome equilibrium.

These findings were corroborated by rank-abundance (Figure 8C) and rarefaction (Figure 8B) curves, which indicated a reduction of both evenness and species richness of the DSS group. Specifically, Shannon, Chao, and Ace indices of the DSS group decreased significantly from those of the control group (P < 0.0001, P < 0.001, P < 0.001, respectively), and the Simpson index was considerably higher than that of the control group (P < 0.0001). Conversely, the LDPL and LDPH groups demonstrated significantly higher Shannon (p < 0.0001 both), Chao (p < 0.01, p < 0.0001), and Ace (p < 0.01, p < 0.0001) indices and lower Simpson index (p < 0.0001 both) compared to the DSS group (Figure 8D). In summary, the result suggests that DSS-induced colitis alters gut microbiome diversity and richness. However, LDP treatment appears to facilitate the restoration of these alpha diversity parameters in a dose-dependent manner. Beta diversity was assessed using NMDS, PCoA, and PCA, which collectively demonstrated structural variations in the bacterial communities across all groups. Highly significant differences were observed for PCA in PC1 (p < 0.0001) and for PCoA (p < 0.0001). Our results revealed that the DSS group samples clustered distinctly from the control group, indicating a substantial shift in microbial structure. In contrast, the LDPH and LDPL groups exhibited community profiles more similar to the control group than to the DSS group (Figure 8E).

Furthermore, hierarchical clustering and heatmap analysis (Figures 8F,G) revealed distinct microbial profiles among the four groups (NC, DSS, LDPL, LDPH). The DSS group formed a separate cluster, highlighting significant disruption of the gut microbiota. Conversely, LDPL and LDPH groups clustered more closely with NC, suggesting that LDP treatment partially restored microbial composition toward a healthy state. The dendrogram further supported these findings, showing clear separation of DSS samples and overlapping clusters among NC, LDPL, and LDPH groups, underscoring the modulatory effects of LDP on DSS-induced dysbiosis.

### 3.11 Structural alterations in gut microbiota at taxonomic levels induced by LDP in an ulcerative colitis murine model

**FIGURE 9:**
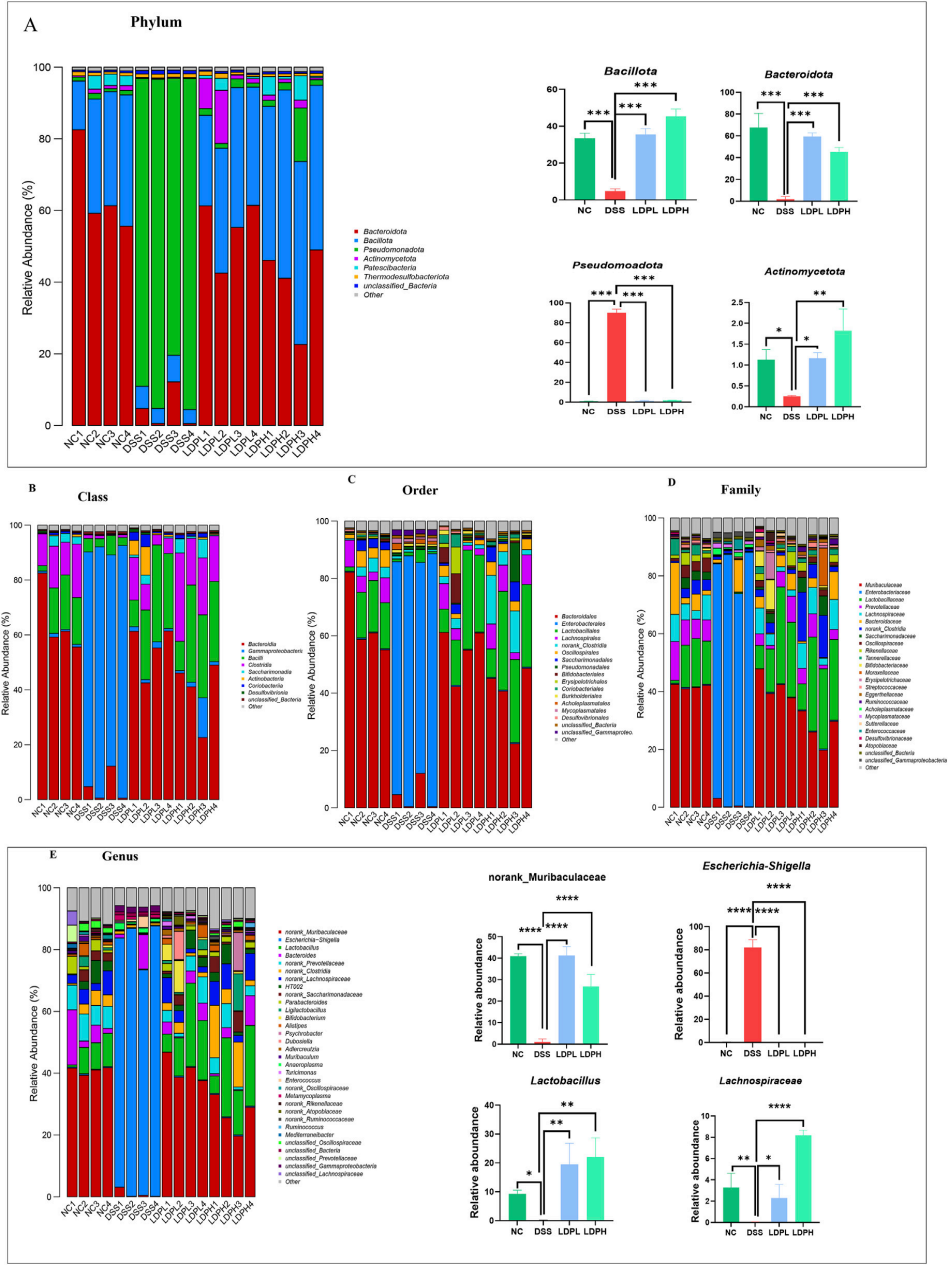
Influence of LDP in the microbiota ecology. **(A)** Structure of microbiota at the phylum level with graphs showing the relative abundance of main phyla in different groups. **(B)** Structure of microbiota at the class level. **(C)** At the order level. **(D)** At the family level. **(E)** Structure of microbiota at the genus level with graphs showing of main phyla in different groups. Data are presented as mean ± SD. Statistical significance was determined by one-way ANOVA with *post hoc* t-tests (*p < 0.05, **p < 0.01, ***p < 0.001, ****p < 0.0001).

**FIGURE 10:**
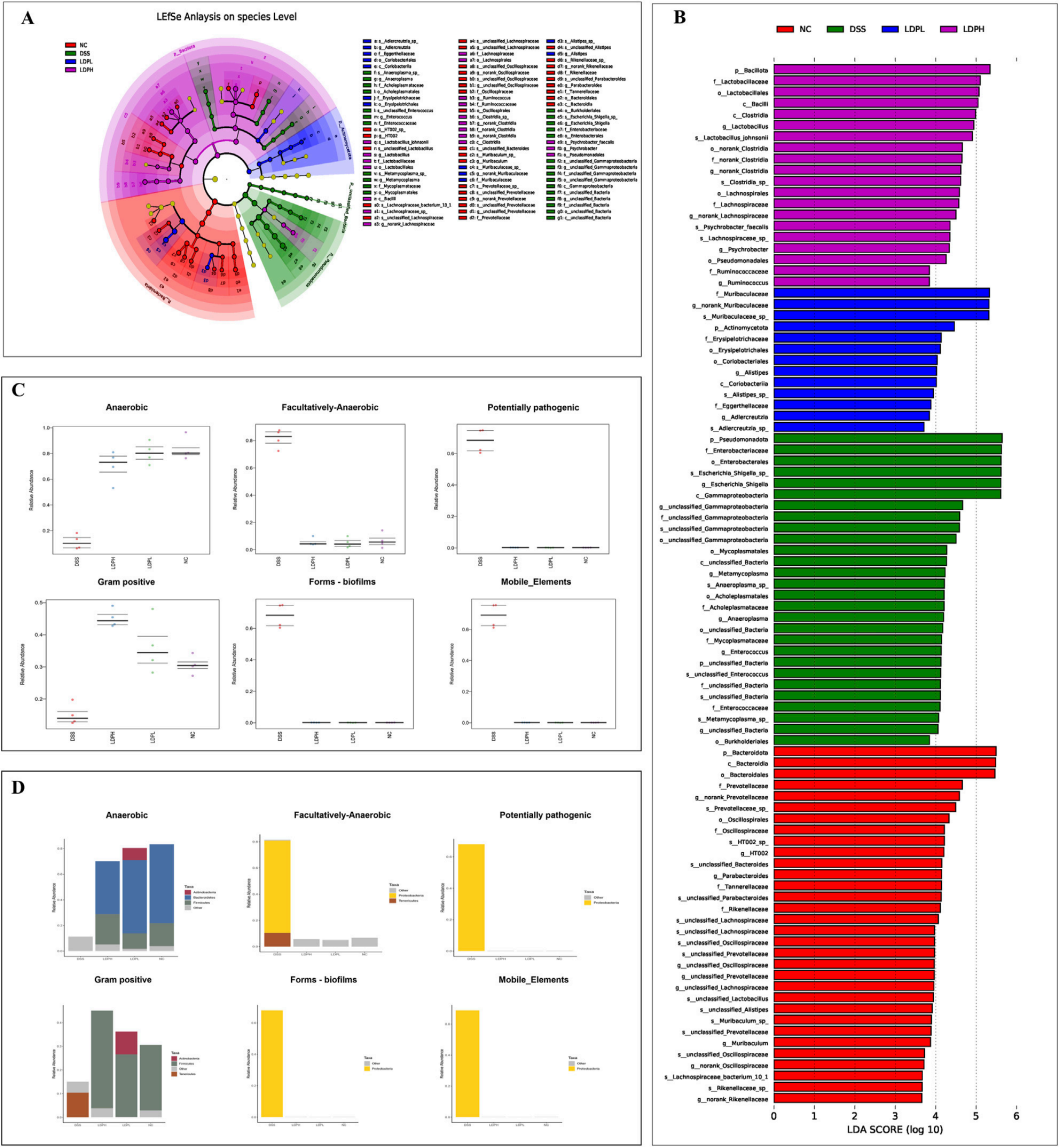
Linear Discriminant Analysis (LDA) effect size (LEfSe) analysis of bacterial taxa and BugBase-predicted phenotype profiling. **(A)** The cladogram depicts the taxonomic distribution of highly abundant microbial taxa across experimental treatment groups. **(B)** Linear Discriminant Analysis (LDA) identified the most differentially abundant bacterial taxa among specific sample groups. **(C)** BugBase identified differentially abundant phenotypes across groups, including anaerobic, facultative anaerobic, pathogenic potential, Gram-positive, biofilm formation, and mobile elements. **(D)** A bar graph displays the relative abundance of each phylum within the various phenotypes.

To evaluate LDP-mediated modulation of colonic microbiota and its restorative efficacy on dysbiosis, gut microbiota was taxonomically characterized (Figures 9A–E). At the phylum level, DSS-induced colitis led to severe dysbiosis of the gut microbiota, as represented by a remarkable decrease in beneficial phyla, including Bacillota, Bacteroidota, and Actinomycetota, with a profound overrepresentation of Pseudomonadota (p < 0.001), which is commonly linked to inflammation and disease progression (Gómez-Zorrilla et al., 2017). LDPL and LDPH treatments successfully resumed microbial homeostasis by promoting a significant enhancement of the relative proportion of commensal Bacillota and Bacteroidota, two pivotal phyla that are associated with gut barrier integrity, short-chain fatty acid production, and anti-inflammation (Van Hul et al., 2024). Specifically, both treatments had a profound inhibition on the pathogenic development of Pseudomonadota (p < 0.001). The increased richness of Actinomycetota, especially in the LDPH group (p < 0.01), is another indicator of the immunomodulatory and restorative properties of the treatment. These findings indicate that LDP ameliorates DSS-induced dysbiosis and promotes a healthier gut microbiota profile. Furthermore, Bacterial taxonomy was analyzed at multiple hierarchical levels, including class, order, family, and genus. The results show that, unlike the normal control group, the DSS group had a higher abundance of the class Gammaproteobacteria, but lower levels of the classes Bacteroidia, Bacilli, and Clostridia. Importantly, LDP supplementation helped restore balance in gut bacteria at both low and high doses, increasing the levels of Bacteroidia, Bacilli, and Clostridia compared to the DSS group (Figure 9B). Besides, in the DSS group, Enterobacterales was the most prevalent order (Figure 9C), whereas Bacteroidales, Lactobacillales, and Lachnospirales were less prevalent than in the normal control group. Conversely, in the treatment groups, there was a remarkable recovery of the microbial imbalance. Bacteroidales, Lactobacillales, and Lachnospirales were the most prevalent bacterial orders. On the family level (Figure 9D), gut microbiota composition analysis revealed remarkable variations in the various experimental groups. In the NC, bacterial gut composition was mostly characterized by the dominance of Muribaculaceae, Lachnospiraceae, and Prevotellaceae, indicating a healthy, balanced gut microbiota. Conversely, the DSS-induced colitis model was characterized by an evident dysbiosis in gut bacteria, corroborated by a high abundance of Enterobacteriaceae and a profound reduction in microbial diversity. Yet, microbial balance was enhanced by both LDP doses, as seen through the recovery of beneficial families such as Lachnospiraceae and Muribaculaceae. Interestingly, the LDPH treatment group had a significantly better recovery with a microbial population that very much resembled the healthy control. The findings show that the high-dose treatment is superior in remitting DSS-induced dysbiosis and restoring the balance of the gut microbiome to its original status. At the genus level (Figure 9E), DSS-induced colitis led to drastic changes in the structure of the gut microbiota. *Norank_Muribaculaceae* was the most prevalent constituent of the microbiota in the NC group; however, its predominance significantly decreased in the DSS group (P < 0.0001). On the other hand, the *Escherichia-Shigella* abundance was significantly greater in the DSS group than in the NC group, which implicates a potentially detrimental alteration with an inflammatory state. LDPL and LDPH treatments both caused elevated levels of beneficial bacterial groups like *norank_Muribaculaceae* and *Lactobacillus*, indicating a noteworthy trend in the DSS group. Besides, *norank_Lachnospiraceae*, a gut health-related bacterial population and short-chain fatty acid producer, presented a remarkable enhancement after high-dose treatment of LDP compared to the DSS and LDPL groups (P < 0.0001). This suggested that the intervention had the potential to counteract DSS-induced dysbiosis and promote the growth of beneficial gut microbiota. Moreover, LEfSe was utilized to discover the taxonomic differences present among microbial populations in the experimental groups. The results are presented in Figures 10A,B, which indicate taxa with statistical variation among the experimental groups. The NC group exhibited higher variability of bacteria associated with a healthy gut, such as Bacteroidota, Prevotellaceae, and *Bacteroides*. Several previously undescribed and new bacterial taxa, such as *s_HT002* and *norank_Oscillospiraceae,* occurred in higher frequencies, which points to the normal gut microbiota diversity and complexity. Conversely, the DSS group was characterized by a high abundance of pathogenic and potentially toxic bacteria, including Pseudomonadota, *Escherichia/Shigella,* Enterobacteriaceae, and unclassified Gammaproteobacteria, characteristic of complications in colitis models. This overgrowth is a hallmark feature of dysbiosis in the DSS group. The LDPL group had higher levels of certain bacteria like Muribaculaceae, *Ruminococcus*, and *Coriobacteriales*, suggesting partial improvement in microbial imbalance. However, the most significant improvement was observed in the LDPH group, which had a greater diversity of bacteria, primarily from the Firmicutes phylum, including Bacillota, Lactobacillaceae, *Lactobacillus*, and *Lactobacillus johnsonii*, indicating that beneficial bacteria returned with the high-dose treatment. These findings suggest a potential probiotic effect of the high-dose LDP extract. Within the context of DSS treatment, the gut microbiome was significantly enriched for functional properties of dysbiosis, which encompassed anaerobiosis, facultative anaerobiosis, pathogenicity, biofilm production, and mobile genetic elements that were all higher than in the treatment and NC groups. Moreover, Functional characterization of gut microbiota of the NC, DSS, LDPL, and LDPH groups shows a deep dysbiosis induced by the treatment with DSS, as shown by high prevalences of anaerobes and facultatively anaerobic bacteria, potential pathogenic taxa, biofilm-forming bacteria, and mobile genetic elements (Figures 10C,D). The perturbations noted were mainly due to an overrepresentation of Proteobacteria and other inflammation-related taxa. Conversely, both polysaccharide-treated groups, LDPL and LDPH, had microbial communities that more closely resembled the NC group, with decreased pathogenic potential and increased functional diversity. These results reinforce the therapeutic potential of the intervention, particularly at higher doses, in modulating the gut microbiota and potentially alleviating DSS-induced colonic inflammation.

## 4 Discussion

Inflammatory bowel disease encompasses a group of conditions characterized by ulceration of the mucosal and submucosal layers. Among these, ulcerative colitis is one of the most prevalent forms, presenting with superficial inflammation of the colon mucosa. The inflammatory process usually begins in the rectum and gradually spreads to nearby parts of the colon as the severity of the disease worsens (Baumgart and Sandborn, 2012). Commonly, DSS is used as a tool in developing animal models of colitis, due to its ability to accurately mimic the morphological characteristics and clinical symptoms of UC in humans. This model is a useful means of describing the mechanisms of the disease and predicting the potential efficacy of different treatment approaches (Randhawa et al., 2014; Liu et al., 2020). Traditional pharmacological treatment of ulcerative colitis is associated with unwanted side effects and is not relapse-preventing. Hence, it is extremely necessary to search for new natural therapeutic agents in inflammatory bowel disease (Li Z.-Y. et al., 2023). The current research is a pioneering assessment of crude polysaccharides from the *L. decastes* fungal species, a well-known fungus for its antitumor, anti-diabetic, and immunoregulatory potential (Ukawa et al., 2000; Miura et al., 2002; Ma and Wang, 2023). This study selectively examines the use of these polysaccharides to reduce the symptoms of inflammatory reactions associated with ulcerative colitis, i.e., destruction of colonic tissue, dysfunction in the immune system, and alteration in gut microbial flora. Polysaccharides from *L. decastes* were extracted by a hot water method, and the monosaccharide composition was analyzed by HPLC. In agreement with other reports, the predominant components identified were glucose, galactose, mannose, fucose, ribose, and galacturonic acid (Zhang et al., 2023). The result of GPC analysis revealed that the crude LDP polysaccharide possesses a very high molecular weight profile with a weight-average molecular weight (Mw) of approximately 5.59 × 10^6^ g/mol and a number-average molecular weight (Mn) of 2.47 × 10^6^ g/mol. The broad distribution of molecular weights—manifested in several size fractions—suggests a heterogeneous polysaccharide mixture possessing complicated structural characteristics. The presence of high molecular weight and structural heterogeneity is generally associated with increased bioactivities, such as immunomodulatory, antioxidant, and anti-inflammatory activities. A comprehensive review conducted by Wang et al. (2022) reveals that polysaccharides contained in certain high molecular weight ranges possess much higher immunomodulatory and anti-inflammatory activities than their counterparts in lower molecular weight. These findings are consistent with the hypothesis that molecular size is one determinant of polysaccharide bioactivity, most likely by facilitating greater interactions with immune receptors and stabilization of conformational structure pertinent to biological activity. In this study, a model of ulcerative colitis was induced by treatment with 3% DSS in drinking water for 7 days. This resulted in a significant decrease in body weight and clinical signs such as rectal bleeding, diarrhea, and a change in stool consistency compared to the untreated controls. A reduction in the length of the colon was also observed, with the implication of a correlation with the degree of inflammation and the development of ulcerative colitis. However, oral administration of LDP showed a protective effect, mitigating these clinical signs; the result is consistent with previous findings by Kanwal et al. (2020), which reported similar protective effects of mushroom-derived polysaccharides in DSS-treated mice. In their study, fungal polysaccharides attenuated body weight loss, preserved colon length, and improved histological features of the colon. Our results align closely with these observations, highlighting the comparable therapeutic potential of LDP in modulating inflammatory responses and preserving intestinal structure in UC models. Furthermore, similar improvements were observed in body weight, colon length, and other clinical indicators following LDP treatment. The thymus and spleen are essential lymphoid organs responsible for the development and regulation of immune responses (Liu et al., 2022). And changes in their size reflect an immune system imbalance. Inflammatory conditions such as DSS-induced colitis are often associated with thymic atrophy and splenic hypertrophy, indicative of immune dysfunction. In our study, DSS administration significantly reduced the thymic index and increased the splenic index, as well as decreased colon and small intestine indices, confirming systemic inflammation. However, LDP treatment markedly reversed these changes, suggesting its potential immunoregulatory role. These results are consistent with those reported by Li et al. (2022), which demonstrated that *Ganoderma lucidum* polysaccharides restored the thymus and spleen indices and improved gut histology in a similar colitis model.

The integrity of the intestinal barrier plays a pivotal role in maintaining gastrointestinal homeostasis and protecting the host from harmful luminal contents. It is regulated by tight junction proteins such as occludin and ZO-1, which are critical for controlling paracellular permeability (Fortea et al., 2021; Di Vincenzo et al., 2024). This particular barrier defect has often been termed ‘leaky gut’ and has been implicated in numerous disorders of the digestive system and other systems of the body (Bischoff et al., 2014). In our study, DSS administration led to significant disruption of this barrier, evidenced by histological damage, reduced goblet cell numbers, and downregulation of TJ proteins. Findings consistent with previous reports (Chassaing et al., 2014; Ghosh et al., 2021; Gou et al., 2022). Remarkably, treatment with LDP, particularly at high doses, restored epithelial integrity and upregulated occludin and ZO-1 expression in a dose-dependent manner. Similar protective effects on TJ proteins have been reported with polysaccharides from *Allium sativum* L. (Shao et al., 2020) and *Cantharellus cibarius* Fr. (Alioui et al., 2024); however, the degree of barrier restoration observed with LDP was more pronounced. These findings suggest that LDP may enhance epithelial resilience through unique mechanisms, reinforcing its potential as a mucosal-protective agent in UC management.

MPO is a commonly used inflammatory marker to assess the extent of inflammatory cell infiltration and determine the degree of tissue damage. An increase in MPO concentration is associated with ulcerative colitis (Ren et al., 2018; Cartwright et al., 2024). Our results showed a significant increase in MPO levels in the group treated with DSS only, compared to the control group. However, treatment with LDP significantly reduced MPO levels in a dose-dependent manner. This result is consistent with that reported in (Hu et al., 2024), which shows the ability of *Phellinus linteus* polysaccharide to reduce MPO levels in colon tissue.

Inflammation affecting the lining of the intestine disrupts the delicate equilibrium between the antigens within the gut and the immune response of the organism. This imbalance leads to impairment of the gut’s physical protective layer, enabling detrimental bacteria and their byproducts from metabolism to activate the immune system. Consequently, this triggers the secretion of cytokines that promote inflammation, such as IL-1β, IL-6, and TNF-α, which subsequently amplify the inflammatory reaction. This dysregulation in the balance between pro-inflammatory and anti-inflammatory cytokines worsens uncontrolled inflammation in the intestines, causing further harm to the intestinal mucosal barrier and disturbing the composition of the intestinal microbiota. These factors are crucial in the development of ulcerative colitis (Peng et al., 2024). The immunomodulatory activity observed in our study, particularly the significant suppression of pro-inflammatory cytokines (TNF-α, IL-1β, IL-6) and the concurrent upregulation of anti-inflammatory markers (IL-10, IL-4, TGF-β), is consistent with findings reported by Shao et al. (2020), who evaluated *Allium sativum* L. polysaccharide in a DSS-induced colitis model, and also in agreement with (Sun et al., 2024), which reported the ability of *L. decastes* polysaccharides to downregulate the expression level of TNF-α and IL-6. However, unlike their study, which primarily focused on downregulation of TNF-α and IL-6, our results also demonstrated robust elevation of TGF-β and IL-4, indicating a more comprehensive shift toward anti-inflammatory immune regulation.

Over the last decade, the role played by the intestinal microbial community in the development of UC has garnered progressively more scientific focus. Under typical physiological conditions, the microbiota exists in a condition of ongoing balance, carrying out crucial functions such as the breakdown of non-digestible carbohydrates via fermentation, the creation of short-chain fatty acids (SCFAs), energy production, the generation of necessary vitamins, defense against disease-causing organisms, and the upkeep of the intestinal lining’s structural soundness (Khan et al., 2019; Alsholi et al., 2023). An imbalance in intestinal microbiota can result in immune dysfunction and is linked to the development of UC (Wu et al., 2018; Wang et al., 2022b). During UC treatment, regulating intestinal microbiota helps repair the intestinal mucosa, enhances the inflammatory response, and boosts patients' immunity, which can aid in alleviating or even curing the disease (Guo et al., 2020). Therefore, we hypothesized that LDP polysaccharide treatment could alleviate UC by regulating the composition and enhancing the diversity of the microbiota.

In this research, we analyzed the composition of the intestinal microbiome using 16S rRNA sequencing technology on the Illumina MiSeq platform. Consistent with previous studies (Munyaka et al., 2016; Marius et al., 2024; Orejudo et al., 2024), which reported a decrease in microbial diversity and richness in ulcerative colitis patients and animal models, we observed a significant decrease in both abundance and diversity of gut microbiota in the DSS treatment group compared to the control group. Notably, LDP treatment restored both indices. Beta-diversity analyses also revealed a clear variation in clustering patterns, with the LDP-treated group converging with the control group, while the DSS group had a distinct microbial distribution pattern. Venn diagram and alpha diversity analyses revealed a significant reduction in OTU richness in the DSS-induced colitis group, consistent with the known dysbiosis associated with intestinal inflammation. Interestingly, treatment with LDP partially restored microbial richness in the low-dose group, while the LDPH exhibited even higher OTU counts than the healthy control, indicating a strong microbiota-enhancing effect. One of the most striking findings of this study was the significant increase in gut microbial richness observed in the LDPH group, which even surpassed that of the normal control group. This suggests that LDP may not only reverse DSS-induced dysbiosis but also promote a supraphysiological restoration of microbial diversity. Previous studies with mushroom-derived polysaccharides, such as those from *Phellinus linteus* (Hu et al., 2024) and *Hericium erinaceus* (Ren et al., 2023), have demonstrated beneficial effects in restoring microbial balance in ulcerative colitis models. However, none have reported such a pronounced increase in OTU richness beyond baseline levels. This highlights a potentially unique mechanism of action for LDP, which may be related to its specific monosaccharide composition or structural features that selectively favor the growth of beneficial bacterial taxa, such as Muribaculaceae and Lachnospiraceae. These findings support the novel microbiota-modulating potential of LDP as a promising therapeutic strategy for UC.

To identify differences in microbial composition between different groups, we studied intestinal microbial communities at multiple taxonomic levels. The main phyla of these communities were found to be Firmicutes bacteria, Bacteroidetes, and Proteobacteria. Previous research suggests that dysbiosis is a hallmark of IBD, disrupting the normal balance and composition of the microbial community. These changes include a decrease in the relative abundance of both Firmicutes and Bacteroidetes bacteria, and an increase in the proportion of Proteobacteria (Frank et al., 2011; Son et al., 2019). In line with these findings, our results revealed a significant increase in the relative abundance of Proteobacteria and a reduction in Firmicutes in the DSS-only group. Notably, treatment with LDP effectively reversed these dysbiotic changes, restoring the microbial profile at the phylum level to a composition similar to that of the healthy control group. This outcome is consistent with the findings of Cui et al. (2021), which reported that a polysaccharide from *Scutellaria baicalensis* also ameliorated DSS-induced colitis by modulating gut microbiota composition. We performed an in-depth analysis of microbial flora with a focus on their taxonomic identity at lower levels. Inflammatory bowel disease is characterized by an imbalance in the composition of the intestinal microbiome, characterized by a significant decrease in diversity and an increase in the proportion of Proteobacteria, particularly Enterobacteriaceae and Bilophila. Experiments have shown that elevated levels of Proteobacteria in the colon are associated with negative effects on the mucin layer and intestinal barrier function in DSS-treated mice, where a decrease in goblet cells, MUC2 production, and increased permeability were observed (Xu et al., 2018; Guo et al., 2022a; Guo et al., 2022b). Consistent with previous findings, our results revealed a significant increase in the abundance of Enterobacteriaceae in the DSS group, which was markedly attenuated following LDP intervention. Notably, the DSS-induced colitis model also showed a remarkable upsurge in *Escherichia-Shigella* at the genus level, a hallmark of dysbiosis associated with inflammation (Jialing et al., 2020). After treatment with LDP, this increase was significantly reduced to levels comparable to the healthy control group. These findings are in line with previous reports demonstrating that *Dictyophora indusiata* polysaccharides can alleviate colitis by suppressing *Escherichia-Shigella* abundance and restoring microbial balance (Kanwal et al., 2020). Meanwhile, at the genus level, intestinal symbiotic bacteria like *nanorank-Muribaculaceae* and *nanorank-Lachnospiraceae* rose in the LDP treatment groups and were negatively correlated with IBD development. The *Muribaculaceae* population has a propionate-producing fermentation pathway, and the relative expansion of *Muribaculaceae* abundance in the probiotic group was linked to a rise in propionate concentration. *Lachnospiraceae*, a key member of gut microbiota, consistently expands in species richness and relative abundance throughout the life of the host, and it is one of the significant producers of SCFAs (Li et al., 2024). Probiotics, i.e., *Lactobacillus* and *Bifidobacteria* strains, are effective in the prevention and treatment of IBD because they possess useful properties. These properties are their capabilities to adhere to intestinal epithelial cells, improve immune function, display antimicrobial activity, and diminish metabolic-related disorders (Li C. et al., 2023). Our findings indicate a noteworthy reduction in the population of these helpful bacteria, i.e., Muribaculaceae, Lachnospiraceae, *Lactobacillus*, and *Bifidobacteria*, in the DSS-treated group as compared to the normal control, but LDP appears to restore the dysbiosis. The modulation of gut microbiota observed in our study mirrors findings by Tao et al. (2017), who reported that *Chrysanthemum morifolium* polysaccharide ameliorates colitis by the expansion of beneficial bacteria such as *Lactobacillus* and *Bifidobacteria*. Interestingly, our results extend this concept by highlighting the enrichment of Muribaculaceae and Lachnospiraceae, bacterial families known for their short-chain fatty acid production and immunomodulatory properties. These observations imply that LDP supports a more diverse and functional microbial recovery, potentially enhancing host–microbiota interactions during colitis resolution. Moreover, Functional characterization of gut microbiota of the NC, DSS, LDPL, and LDPH groups shows a deep dysbiosis induced by the treatment with DSS, as shown by high prevalence of anaerobes and facultatively anaerobic bacteria, potential pathogenic taxa, biofilm-forming bacteria, and mobile genetic elements. The perturbations were mainly due to an overrepresentation of Proteobacteria and other inflammation-related taxa. Conversely, both polysaccharide-treated groups, LDPL and LDPH, had microbial communities that more closely resembled the NC group, with decreased pathogenic potential and increased functional diversity. These results align with previous work done by Kanwal et al. (2020). These results reinforce the therapeutic potential of the LDP intervention in modulating the gut microbiota and potentially alleviating DSS-induced colonic inflammation.

The comprehensive analysis of our data across all taxonomic levels consistently demonstrated a clear shift in the bacterial composition following treatment with DSS alone. However, and crucially, the administration of LDP at both high and low doses significantly reversed this DSS-induced bacterial dysbiosis. These observations strongly suggest that LDP possesses immunomodulatory properties, likely influencing the gut microbiota and mitigating the disruptive effects of DSS.

Taken together, these findings not only reinforce the therapeutic relevance of LDP in colitis but also underscore several novel contributions of this study. To the best of our knowledge, this is the first investigation to evaluate the therapeutic potential of polysaccharides derived from *L. decastes* in the context of ulcerative colitis. While numerous studies have explored polysaccharides from other edible and medicinal fungi, the biological activity of LDP in modulating gut inflammation and microbiota composition remains uncharted. This study uniquely integrates multi-dimensional assessments—including histopathological analysis, tight junction protein expression, cytokine profiling, and gut microbiota sequencing—to demonstrate the comprehensive protective effects of LDP against DSS-induced colitis. Moreover, the results highlight LDP’s capability to restore microbial diversity, enhance beneficial bacterial taxa, and suppress pathogenic populations, offering new insights into its immunomodulatory and microbiota-regulating properties. These findings open promising avenues for the ethnopharmacological application and functional food development of *L. decastes* as a natural therapeutic candidate for ulcerative colitis management.

## 5 Conclusion

This study highlights the novel therapeutic potential of LDP in the treatment of ulcerative colitis. The findings reveal that LDP polysaccharides exhibit substantial protective effects by significantly alleviating inflammatory symptoms and improving critical physiological parameters such as body weight, food, and water intake. Notably, LDP promotes the induction of goblet cells and mucin secretion, alongside the upregulation of tight junction proteins, suggesting a restorative effect on intestinal barrier integrity. In addition to its physical effects, LDP demonstrates a potent immunomodulatory action, enhancing anti-inflammatory cytokine levels while suppressing pro-inflammatory cytokines. Furthermore, LDP contributes to the restoration of gut microbial homeostasis by fostering the growth of beneficial commensal bacteria and inhibiting pathogenic species. In conclusion, *L. decastes* polysaccharide demonstrated significant protective effects against DSS-induced colitis in mice. While further studies are needed, including clinical evaluation and mechanistic exploration, these findings suggest that LDP holds promise as a natural microbiota-modulating and anti-inflammatory candidate for ulcerative colitis management.

## Data Availability

All data presented in the study are included in the article/Supplementary Material, further inquiries can be directed to the corresponding authors.
