# Supplementary material for: Lyophyllum decastes-derived polysaccharides alleviate DSS-induced colitis in mice by suppressing inflammation, enhancing intestinal barrier integrity, and restoring gut microbiota homeostasis
Source: Front Pharmacol. 2025 Jul 11;16:1644325. doi: 10.3389/fphar.2025.1644325 (PMC12289643; doi:10.3389/fphar.2025.1644325)
Supplement: Supplementary file 1 [file Table1.docx]

*Lyophyllum decastes-*Derived Polysaccharides Alleviate DSS-Induced Colitis in Mice by Suppressing Inflammation, Enhancing Intestinal Barrier Integrity, and Restoring Gut Microbiota Homeostasis

**Eslam Fahd^1^, Yamina Alioui^2^, Jamalat Yazeed^3^, Natheer Wahan^3^, Ahmed Bashah^4^, Ali AL-waqeerah^4^, Aamna Atta^1^, Yuxin Sun^2^, Yi Xin^2^, Liang Wang^5^*, Bin Feng^1^*, Weifeng Mao^2^***

^1^Biochemistry and molecular biology department, College of Basic Medical Sciences, Dalian Medical University, Dalian 116044, China

^2^ Bitechnology department College of Basic Medical Sciences, Dalian Medical University, Dalian 116044, China

^3^ College of Pharmacy, Dalian Medical University, Dalian 116044, China

^4^ The First Affiliated Hospital of Dalian Medical University, Dalian 116011, China

**^5^** Stem Cell Clinical Research Center, National Joint Engineering Laboratory, Regenerative Medicine Center, The First Affiliated Hospital of Dalian Medical University, Dalian 116011, China

*** Correspondence:**Corresponding Author
E-mail addresses: wangliang@dmu.edu.cn (Liang Wang), binfeng@dmu.edu.cn (Bin Feng), maoweife@hotmail.com (Weifeng Mao).

# Extracted Supplementary Tables

## Table S1. Antibodies used in IHC and IF

| Antibody target | Antibody type | Antibody dilution | Catalog Number | Company |
| --- | --- | --- | --- | --- |
| Mucin-2 | Polyclonal | 1:1000 | 27675-1-AP | Proteintech |
| ZO-1 | Polyclonal | 1:1000 | 21773-1-AP | Proteintech |
| Occludin | Polyclonal | 1:400 | 27260-1-AP | Proteintech |

## Table S2. List of primer sequences (5' to 3’) employed for assessing mRNA expression level

| Gene | Forward primer | Reverse primer | Concentration | Supplier |
| --- | --- | --- | --- | --- |
| IL-6 | TACCACTTCACAAGTCGGAGGC | CTGCAAGTGCATCATCGTTGTTC | 10 µM | Thermo Fisher Scientific |
| IL-10 | CGGGAAGACAATAACTGCACCC | CGGTTAGCAGTATGTTGTCCAGC | 10 µM | Thermo Fisher Scientific |
| TNF-α | GGTGCCTATGTCTCAGCCTCTT | GCCATAGAACTGATGAGAGGGAG | 10 µM | Thermo Fisher Scientific |
| TGF-B | TGGAGCAACATGTGGAACTC | GTCAGCAGCCGGTTACCAAG | 10 µM | Thermo Fisher Scientific |
| β-Actin | ATCGCTGCGCTGGTCG | GTCCTTCTGACCCATTCCCA | 10 µM | Thermo Fisher Scientific |
